# Cognitive ability, education, height and body mass index in relation to risk of schizophrenia and mortality following its diagnosis

**DOI:** 10.1007/s10654-024-01140-6

**Published:** 2024-07-27

**Authors:** Terese Sara Høj Jørgensen, Ida Kim Wium-Andersen, Marie Kim Wium-Andersen, Maarten Pieter Rozing, Martin Balslev Jørgensen, Thorkild IA Sørensen, Merete Osler

**Affiliations:** 1 Center for Clinical Research and Prevention, Bispebjerg and Frederiksberg Hospitals, Nordre Fasanvej 57, 2000 Frederiksberg, Denmark; 2 Section of Social Medicine, Department of Public Health, University of Copenhagen, Øster Farimagsgade 5, K 1014 København, Denmark; 3 Department of Public Health, The Research Unit for General Practice and Section of General Practice, University of Copenhagen, Øster Farimagsgade 5, Copenhagen, K 1014 Denmark; 4 Psychiatric Centre Copenhagen dept O, Rigshospitalet, Edel Sauntes Allé 10, Copenhagen, 2100 Denmark; 5 Section of Epidemiology, Department of Public Health, University of Copenhagen, Øster Farimagsgade 5, Copenhagen, K 1014 Denmark; 6 Novo Nordisk Foundation Center for Basic Metabolic Research, Faculty of Health and Medical Sciences, University of Copenhagen, Copenhagen, Denmark

**Keywords:** Schizophrenia, Psychiatric disease, Mortality, Traits in young adulthood, Epidemiology, Men

## Abstract

This study examines the hypotheses that the traits of higher IQ, longer education and taller height are associated with lower risk of death as compared to traits of low IQ, short education, and short height in men with schizophrenia compared to men without schizophrenia. In total, 937,919 men born 1939-59 and 1983–1997 with information from conscription were followed for incident schizophrenia in Danish registries. Higher levels of cognitive ability, longer education, and taller height were associated with fewer cases of schizophrenia. In a sub-sample of 652,368 men with information on body mass index, underweight was associated with more and overweight and obesity were associated with fewer cases of schizophrenia compared with normal weight. Higher cognitive ability, longer education, and taller height were associated with fewer deaths from both natural and unnatural causes in both men with and without schizophrenia. Underweight was associated with more deaths from natural and unnatural causes, whereas overweight and obesity were associated with more deaths from natural causes and fewer deaths from unnatural causes in both groups of men. Due to interaction, tall height and long educational duration were associated with fewer deaths from natural causes, and obesity was associated with fewer deaths from unnatural causes among men with schizophrenia compared to men without. In conclusion, traits in young adulthood are associated with higher mortality in men with and without schizophrenia, but traits of long educational duration and obesity seem to be especially important for lower mortality in men with schizophrenia.

**Supplementary Information:**

The online version contains supplementary material available at 10.1007/s10654-024-01140-6.

## Introduction

Schizophrenia is a severe mental disorder, where early life biological, psychological and social exposures, which may lead to later traits, have been suggested to play an important aetiological role in addition to the genetic contributions to the disease [1]. A number of previous studies have identified traits such as low cognitive ability, social traits such as poor achievement in primary school and biological traits such as short height, and underweight in young adulthood as risk factors for schizophrenia [2–8].

Individuals with schizophrenia experience on average 11–13 years shorter life expectancy than individuals without schizophrenia [9, 10]. Previous studies have shown that the higher relative risk is especially pronounced for death from unnatural causes [9–11]. The excess mortality of natural causes has been attributed to unhealthy lifestyle, side effects from treatment, and somatic comorbidity in patients with schizophrenia [2, 9].

It is, however, to our knowledge unknown whether traits, which are shaped in early life and identified as important for mortality throughout life in the general population, [12, 13], impose a different risk of death in individuals with and without schizophrenia.

The theory of differential vulnerability describes that socially disadvanced groups often are exposed to many different risk factors at the same time, which makes them more vulnerable to developing poor health outcomes and die [14] Likewise, it is thinkable that individuals with schizophrenia will experience greater risk of death associated with traits such as low cognitive ability, short education, short height, and underweight in young adulthood than individuals without schizophrenia. On the other side, the impact of these traits may also be less pronounced in individuals with schizophrenia because of the large elevated risk of death related to the disease itself [9].

Whether traits of cognitive ability (IQ), educational duration, height and body mass index (BMI) are related to mortality differently in individuals with schizophrenia could help elucidate why this patient group has a materially shorter life expectancy than the general population. This would be a first step leading to future studies that explore the underlying causal mechanisms of possible association.

The first aim was to investigate whether each of the previously identified associations of height, IQ, BMI, and educational duration with incidence of schizophrenia could be replicated in a cohort of Danish men. We hypothesize that each of the traits of higher IQ, longer educational duration and taller height are associated with a lower incidence of schizophrenia than traits of low IQ, short educational duration and short height, respectively. We hypothesize that underweight, overweight or obesity are associated with higher incidence of schizophrenia than normal weight. The second aim was to explore the associations of these traits with subsequent mortality by comparing men with and without schizophrenia. The null hypotheses in these analyses are that each of the traits exhibits the same association with mortality (on an additive scale) in men with and without schizophrenia. The alternative hypotheses are that the associations differed between the two groups, possibly indicating that the presence of schizophrenia may either strengthen or weaken the associations.

## Materials and methods

### Participants

This study was based on data from the Danish Conscription Database (DCD) [15] and the Danish Conscription Registry (DCR) including nearly all Danish men examined at conscription from 1957 to 1984 and 2006–2015, respectively [16]. The data were restricted to men because DCD and DCR only include < 1% women, which are highly selected. Information on the included traits is not available in other Danish registers. The majority of men, especially in DCD, were ethnic Danes. There was a gap in the registration and digitalization of information collected at the Danish conscriptions in the period 1985–2005. In Denmark, all men are requested by law to appear before the conscription board for a health examination between the ages of 18 and 26 years. We linked these data through the Danish national identification number, assigned to everyone living in Denmark since 1968, with the Danish Civil Registration System, the Danish Psychiatric Central Register, the Danish National Patient Registry, and the Danish Cause of Death Registry (Supplementary Fig. 1). The initial study population included 1,092,823 men from DCD and DCR as described in Supplementary Fig. 2. Due to exclusion of 132,151 men with missing information, 8,545 men not in the birth cohort 1939-59 for DCD or not in the age range 18–30 years at the time of conscription, 13,458 females and 750 who were inactive in the CPR register, the study population at risk of death included 937,919 men. Among men excluded from the study due to missing information, the majority had been exempted from the conscription examination due to medical conditions such as mental retardation and other psychiatric conditions, asthma, epilepsy, or type 1 diabetes, however, the specific cause was not available in the register. After exclusion of 564 men with schizophrenia at baseline, the study population at risk of schizophrenia included 937,355 men, and after further exclusion of 285,551 men without measures of BMI, a sub-study population with measures of BMI included 652,368 men (Supplementary Fig. 2). In previous a paper, we have shown that men with missing information on BMI do not represent a selective part of the study population [12]. This information has been added to Supplementary Fig. 2.

The study was approved by the Danish Data Protection Agency. All data were retrieved from administrative registers, which according to Danish law implies that informed consent was not required of participants.

### Measures

The selected traits were measured by cognitive ability, educational duration, height, and body mass index (BMI) in young adulthood identified in DCD and DCR.

Cognitive ability was assessed by the conscription intelligence test called the ‘Børge Prien Prøve’ (BPP), which comprises logical, verbal, numerical, and spatial subtests. The BPP score ranges from 0 to 78 and correlates well with the full-scale Wechsler Adult Intelligence Scale IQ score (*r* = 0.82) [17]. Cognitive ability was investigated by tertiles of birth cohort-specific z-scores. Information on the educational duration reported at conscription was categorized into low (7th -9th grade), medium (vocational training or 10th -11th grade), and high education (12th grade or more advanced).

Information on the height and weight measured at the conscription examination was retrieved from the DCD and DCR databases. Height was measured without shoes and weight was measured wearing only underwear. Height was investigated as tertiles of birth-specific z-scores and BMI (kg/m^2^) was divided into: (i) underweight:<18.5 kg/m^2^, (ii) normal weight:18.5 to < 25 kg/m^2^, (iii) overweight:25 to < 30 kg/m^2^, and (iv) obese:>=30 kg/m^2^ [18]. The lowest tertile of cognitive ability and height and shortest education, respectively, were used as reference to illustrate the possible linear associations. For BMI, normal weight was used as reference category to follow the suggestions by WHO.

#### Schizophrenia

Using the person identification number as a key, schizophrenia was ascertained by the linkage with the Danish Psychiatric Central Register and the Danish National Patient Registry as the first discharge diagnosis from a psychiatric or somatic ward until the end of follow-up (31st of December 2014) [19]. The registers hold individual-level data on the type of patient contact (inpatient information from 1969 to 1977, respectively; emergency room and outpatient information from 1995), discharge diagnosis, and date of admission and discharge for all hospital admissions in Denmark. Diagnoses have been coded according to the 8th Revision of the International Classification of Diseases (ICD-8) from 1969 to 1994, and the 10th Revision (ICD-10) from 1995 and onwards. Schizophrenia was identified by ICD-8: 8:295.x9, 297.x9, 298.29-298.99, 299.04,299.05, 299.09; and ICD-10:F20-F29. ICD-9 was not used in Denmark. There exists a relatively good concordance between the codes used for schizophrenia in ICD-8 and ICD-10 and we used the approach used in previous Danish studies [20].

#### Vital status and causes of mortality

Information on the time of emigration and death was obtained from the Danish Civil Registration System. Information on mortality from natural and unnatural causes was retrieved from the Danish Cause of Death Registry from 1st of January 1970 until 31st of December 2014. Mode of death and specific primary cause of death were used to identify death from unnatural causes. Death from unnatural causes was defined by death of suicide, homicide accidents, and other external causes in the International Statistical Classification of Disease, injury and cause of death. Death from natural causes comprised all other deaths.

### Statistical analysis

In the analytical models with schizophrenia as the outcome, the men were followed from their age on January 1st, 1969 (the date the Danish Psychiatric Central Register was initiated) or date of conscription examination, whichever came last, and until the first registration of a schizophrenia diagnosis, emigration, death or end of follow-up (31st of December 2014), whichever came first. The analytical models for mortality outcomes followed men from April 1st, 1968 (the date the Danish Civil Registration System was initiated) or date of conscription examination, whichever came last, and until the first registration of emigration, death, or end of follow-up (31st of December 2014), whichever came first. Age was the underlying time scale of the models and thus, automatically accounted for.

Nelson-Aalen cumulative hazard curves of schizophrenia, death from natural causes, and death from unnatural causes were calculated taking competing risk of death and death from other causes into account as censoring events. The analyses were stratified by each of the four traits (cognitive ability, educational duration, height, and BMI). The competing risk analysis is not appropriate for time-varying exposures (in this case schizophrenia). Thus, for the competing risk analyses of death from natural and unnatural causes, respectively, comparisons were based on men with schizophrenia and a time-matched reference population. The reference population was randomly selected among members in the cohort who had remained free from schizophrenia at the time of matching and who had reached the same age as the case in a 1:5 ratio (*n* = 73,872). Individuals in the reference group were censored if they were diagnosed with schizophrenia (*n* = 517).

To estimate caseloads rather than relative hazards estimated by the usual Cox regression model, we used Aalen’s additive hazard model [21]. It was first applied to estimate additional incident cases of schizophrenia per 100,000 person-years (py) associated with the traits (young adult cognitive ability, educational duration, height, and BMI). Secondly, the model was used to estimate additional cases of death from natural and unnatural causes, respectively, per 100,000 py associated with the traits including interaction terms with schizophrenia. The assumption of the time-constant hazard difference of this additive model was assessed graphically by the cumulative coefficient vs. time plots. The analyses of the traits cognitive ability, educational duration, and height of young adults were conducted with mutual adjustment for the other traits (young adult cognitive ability, educational duration, height). Furthermore, the multiple adjusted analyses of BMI in the sub-study population were also adjusted for young adult cognitive ability, educational duration, and height. All analyses were stratified on birth cohort in seven categories (1939–1944, 1945–1949, 1950–1954, 1955–1959 for the DCD cohort, and 1985–1989, 1990–1994, 1995–1997 for the DCR cohort). Interactions between schizophrenia and each of the traits measured in early life were investigated by including interaction terms in the models of death by natural and unnatural causes, respectively.

In supplementary analyses, separate additive hazard regression analyses for the DCD and the DCR cohort were conducted to identify possible cohort differences. For these analyses, the DCD cohort was restricted to the birth cohorts 1950–1959 and followed from their conscription examination until 1978-80 to ensure comparable follow-up time in the two cohorts. Also, to account for the possible confounding impact of the father’s social status measured by highest earned education, supplementary analyses for the DCR cohort with adjustment for the father’s educational duration were conducted.

Due to multiple testing of the main analyses (21 test based on 4 (cognitive ability, educational duration, height and BMI), 3 outcome (schizophrenia, death from natural and unnatural causes, respectively) and additionally 9 tests of interaction, we applied Bonferroni corrections and divided the confidence interval with 21. Thus, we used a significance level of 0.24% instead of 5% and calculated 99.76% (from now 99%) confidence intervals for the main analyses.

All analyses were performed in Stata-15 and the statistical software program R.

## Results

Baseline Supplementary Table S1 provides the distribution of educational duration, cognitive ability, height, and BMI at conscription in the cohort of all 937,919 men, the 1:5 matched sample of 61,300 men and the 12,882 men diagnosed with schizophrenia. Men who developed schizophrenia had a lower cognitive ability, educational duration, height and BMI at conscription compared with the full cohort of all men. The distribution of the selected traits was comparable in the cohort of all 937,919 men and the 1:5 matched sample of 61,300 men.

During follow-up (median 34 (range: 17;75) years), 12,318 (1.3%), men were diagnosed with schizophrenia corresponding to an incidence of 42 (95% CI: 41;43) cases per 100,000 py during follow-up to a median age of 60 years (interquartile range: 29;68).

During follow-up, a larger proportion of men with schizophrenia died from natural (3,590 (28.3%) and unnatural causes (1,205 (9.5%)) compared to men without schizophrenia (105,156 (11.2%) died from natural and 18,478 (2.0%) died from unnatural causes). The more specific causes of death from unnatural causes were accidents (47.7% in men without versus 24.1% in men with schizophrenia), suicide (42.8% in men without versus 61.4% in men with schizophrenia) and other (9.6% in men without versus 19.5% in men with schizophrenia). Please see Table S2 in the supplementary material for the distributions of death by natural and unnatural causes for men without and with schizophrenia.

### Risk of schizophrenia

**Fig. 1:**
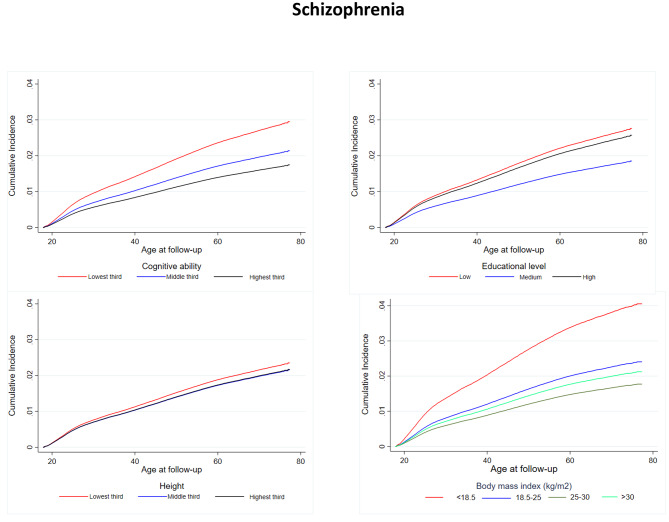
Nelson-Aalen cumulative hazard curves of schizophrenia stratified by young adult cognitive ability, educational level, height, and body mass index

Supplementary Fig. 3 shows the cumulative incidence of schizophrenia, which was 2% at age 60 years. The median age at time of diagnosis for schizophrenia was 37 years (interquartile range: 25; 50). Figure 1 shows the cumulative incidence of schizophrenia stratified by young adult cognitive ability, educational duration, height, and BMI. Men with lower levels of cognitive ability had the highest incidence. Men with a short educational duration had the highest incidence followed by men with high and medium education. Men in the lowest tertile of height had the highest incidence, whereas the incidence was the same for men in the middle and highest tertile. Men with underweight had the highest incidence, whereas men with overweight had the lowest incidence and men with obesity had a slightly higher incidence.

Table 1 presents the results from the additive hazard models and shows that compared with the lowest levels of cognitive ability, shortest educational duration, and shortest height, the medium and the highest/longest/tallest levels were associated with fewer incident cases of schizophrenia per 100.000 py (cognitive ability: -17 (99% CI: -20;-14) and − 20 (99% CI: -23;-17), education: -18 (99% CI: -21;-15) and − 13 (99% CI: -17;-8), height: -4 (99% CI: -6;-2) and − 3 (95% CI: -5;-1)). Underweight was associated with 38 (99% CI: 30;46) more cases, and overweight and obese were associated with − 19 (99% CI: -23;-14) and − 17 (99% CI:-32;-3) fewer cases per 100,000 py than normal weight.

**Table 1:** Additional cases of schizophrenia per 100,000 person-years associated with the traits

		Birth cohort adjusted additional cases of schizophrenia (95% CI)	Mutually* adjusted additional cases of schizophrenia (95% CI)
Full study population (*N* = 937,919)			
Cognitive ability (z-score)	Low	Reference	Reference
	Medium	-22 (-24;-20)	-17 (-19;-15)
	High	-26 (-28;-24)	-20 (-22;-17)
Educational level	Short	Reference	Reference
	Medium	-26 (-28;-24)	-18 (-21;-16)
	Long	-25 (-28;-22)	-12.5 (-16;-9)
Height (z-score)	Low	Reference	Reference
	Medium	-7 (-9;-5)	-4 (-6;-2)
	High	-8 (-10;-6)	-3 (-5;-1)
Study population including BMI measures (*N* = 652,369)			
Body mass index	Underweight	38 (31;44)	38 (32;44)
	Normal	Reference	Reference
	Overweight	-15 (-19;-12)	-19 (-22;-15)
	Obese	-9 (-19;2)	-17 (-28;-7)
*Including all the displayed exposure variables in one model			

### Risk of death from natural causes

For the matched cohort of men, the cumulative incidence of mortality from natural causes at age 70 years was 60% and 20% in men with and without schizophrenia, respectively (Supplementary Fig. 4).

**Fig. 2:**
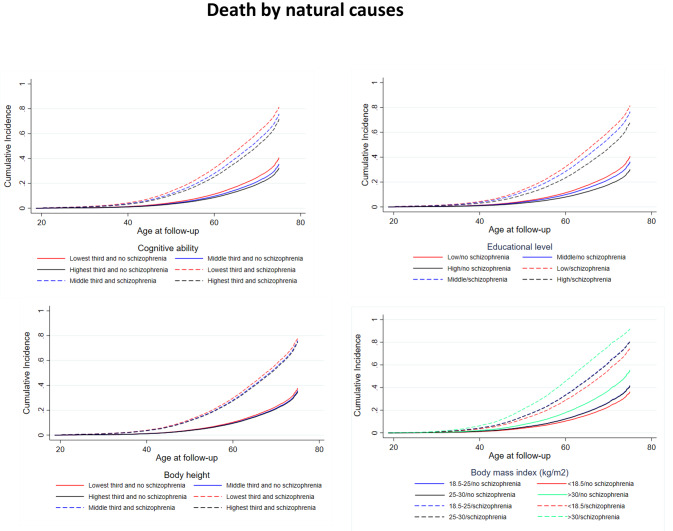
Nelson-Aalen cumulative hazard curves of death from natural causes for men with and without schizophrenia stratified by young adult cognitive ability, educational level, height, and body mass index based on the match cohort of men with and without schizophrenia

Table 2 (two left columns) shows estimates from the additive hazard model of the association between the traits and mortality from natural causes, including interactions between the four traits and schizophrenia per 100,000 py. Men with schizophrenia experienced 1424 (99%CI: 1258; 1589) more deaths than men without. Compared with low cognitive ability, medium and high cognitive ability were followed by -88 (99% CI: -96;-80) and − 142 (99% CI: -152;-131), respectively, fewer deaths. Medium and long educational duration were followed by -66 (99% CI: -74;-57) and − 116 (99% CI: -125;-105), respectively, fewer deaths than short educational duration. Medium and tall height was followed by -37 (99%CI: -44;-30) and − 39 (99% CI: -46;-31), respectively, fewer deaths than short height. Compared with normal weight, underweight (82, 99%CI: 65;99), overweight (66, 99% CI: 50;82) and obesity (187, 99% CI: 143;230) were followed by more deaths. These patterns are also identified in the cumulative incidence curves (Fig. 2).

**Table 2:** Additional deaths per 100,000 person-years associated with the exposure variables for the full study population of men at risk of death

		Additional number deaths from natural cause (95% CI)		Additional number deaths from unnatural causes (95% CI)	
		Birth cohort adjusted	Mutually adjusted*	Birth cohort adjusted	Mutually adjusted*
Full study population (*N* = 937,919)					
Schizophrenia		1312 (1251;1374)	1424 (1298;1550)	532 (498;565)	487 (414;559)
Cognitive ability	Low	Reference	Reference	Reference	Reference
	Medium	-114 (-120;-108)	-88 (-94;-82)	-22 (-24;-19)	-12 (-14;-9)
	High	-198 (-205;-191)	-142 (-149;-134)	-35 (-38;-33)	-17 (-20;-15)
Interaction between cognitive ability and schizophrenia	Low	Reference	Reference	Reference	reference
	Medium	-108 (-242;26)	-7 (-149;135)	64 (-21;149)	59 (-32;151)
	High	-144 (-271;-16)	137 (-27;300)	69 (-18;157)	76 (-32;185)
Educational level	Short	Reference	Reference	Reference	Reference
	Medium	-117 (-123;-111)	-66 (-72;-59)	-36 (-39;-34)	-29 (-32;-27)
	Long	-222 (-230;-214)	-116 (-123;-108)	-51 (-54;-48)	-37 (-40;-34)
Interaction between education and schizophrenia	Short	Reference	Reference	Reference	reference
	Medium	-54 (-180;72)	-76 (-211;60)	73 (-3;150)	51 (-31;133)
	Long	-458 (-604;-312)	-523 (-707;-338)	45 (-52;142)	0 (-119;119)
Height	Low	Reference	Reference	Reference	Reference
	Medium	-59 (-65;-54)	-37 (-42;-31)	-13 (-15;-10)	-8 (-10;-6)
	High	-81 (-86;-75)	-39 (-44;-33)	-19 (-22;-18)	-12 (-14;-9)
Interaction between body height and schizophrenia	Low	Reference	Reference	Reference	Reference
	Medium	-8 (-145;130)	11 (-127;148)	-14 (-99;70)	-22.7 (-108;63)
	High	-184 (-314;-55)	-136 (-267;-6)	-25 (-109;59)	-40 (-129;48)
Study population including BMI measures (*N* = 652,368)					
Schizophrenia		-	1501 (1339;1663)	-	544 (443;645)
Body mass index	Underweight	82 (69;95)	82 (69;95)	16.2 (10;22)	17 (10;23)
	Normal	Reference	Reference	Reference	Reference
	Overweight	87 (75;99)	66 (54;78)	-7 (-11;-3)	-11 (-16;-7)
	Obese	239 (205;272)	187 (154;220)	-4 (-14;7)	-15 (-26;-5)
Interaction between body mass index and schizophrenia	Underweight	58 (-171;288)	43 (-187;274)	53 (-105;211)	53 (-105;211)
	Normal	Reference	Reference	Reference	Reference
	Overweight	317 (-11;645)	292 (-37;620)	-31 (-222;160)	-27 (-217;164)
	Obese	212 (-729;1154)	481 (-765;1120)	-405 (-748;-63)	-401 (-745;-56)
*Including all the displayed exposure variables in one model					

The interaction analyses showed no statistically significant interaction between schizophrenia and the traits with the exception of high educational duration, which were associated with − 523 (99% CI: -765;-280), fewer deaths among men with schizophrenia than men without.

### Risk of death from unnatural causes

For the matched cohort of men, the cumulative incidence of mortality from unnatural causes at age 70 years were 13% and 2% in men with and without schizophrenia, respectively (Supplementary Fig. 4).

**Fig. 3:**
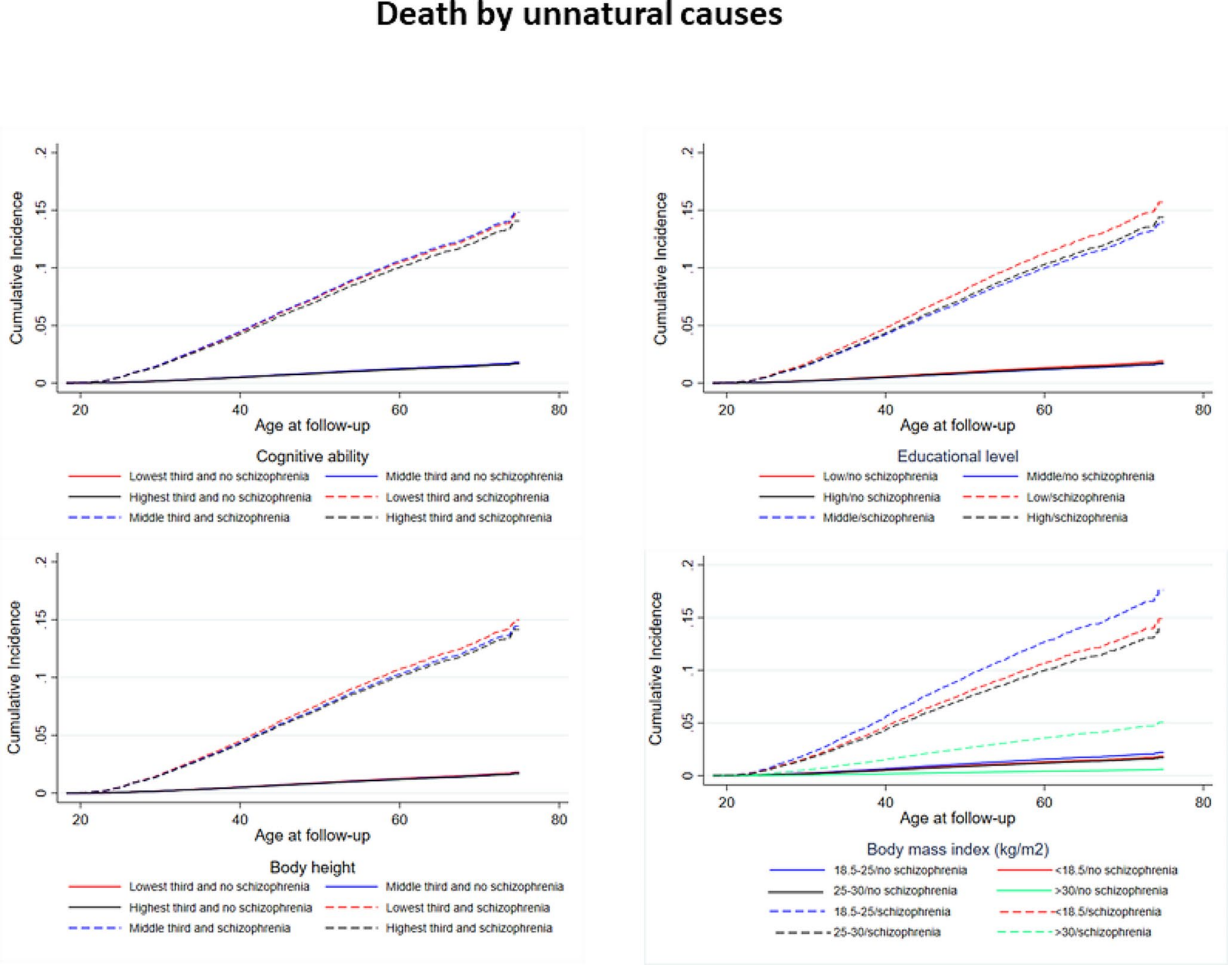
Nelson-Aalen cumulative hazard curves of death from unnatural causes for men with and without schizophrenia stratified by young adult cognitive ability, educational level, height, and body mass index based on the match cohort of men with and without schizophrenia

Table 2 (two right columns) shows estimates from the additive hazard model of the association between the traits and death from unnatural causes. Men with schizophrenia experienced 487 (99% CI: 391;582) more deaths per 100.000 py than men without. Compared with low cognitive ability, medium and high cognitive ability were associated with − 12 (995%CI: -15;-9) and − 17 (99% CI: -21;-14), respectively, fewer deaths. Medium and high educational duration were associated with − 29 (99%CI: -33;-26) and − 37 (99% CI: -41;-32), respectively, fewer deaths than short educational duration. Medium and tall height was associated with − 8 (99%CI: -11;-5) and − 12 (99% CI: -15;-8), respectively, fewer deaths than short height. Compared with normal weight, underweight was associated with 17 (99%CI: 8;25) more deaths, whereas overweight (-11, 99%CI: -17;-6) and obesity (-15, 99%CI:-30;-1) were associated with fewer deaths. These patterns are also apparent in the cumulative incidence curves (Fig. 3).

The interaction analyses showed no statistically significant interaction between schizophrenia and the traits with the exception of obesity, which was associated with − 401 (99% CI: -854;-53) fewer deaths from unnatural causes among men with schizophrenia than men without.

### Supplementary analyses

Supplementary Table S3 and S4 show the supplementary analyses for DCR and DCD of incident schizophrenia and mortality, respectively. Comparisons between the two cohorts showed the same direction of the associations of cognitive ability, educational duration, height and BMI with incident schizophrenia, but with a greater magnitude of the point estimates for the DCR cohort (Supplementary Table S2). The results for deaths from natural causes (Supplementary Table S4) showed that schizophrenia was associated with additional deaths in the DCD cohort (1240, 95%:1070;1400) and, in contrast, schizophrenia was associated with slightly fewer deaths in the DCR cohort (-8, 95%CI: -11;-4). Comparisons between the two cohorts furthermore showed that while the associations of cognitive ability, educational duration, height and BMI with deaths from natural causes were comparable to the main findings for the DCD cohort, the estimates were insignificant for the DCR cohort. Finally, the analyses of deaths from unnatural causes (Supplementary Table S4) showed that schizophrenia was associated with additional deaths in the DCD cohort 556 (95%CI: 448;664), but not in the DCR cohort. The estimates for the associations of cognitive ability and educational duration with deaths from unnatural causes were comparable between the two cohorts, yet, with more pronounced estimates in DCD. Height and BMI were only associated with deaths from unnatural causes in the DCD cohort similar to the main findings.

The results from the analyses of the DCR cohort with adjustment for father’s education (supplementary Table S5 and S6) were comparable to the findings for the DCR cohort without the adjustment (supplementary Table S3 and S4).

## Discussion

In this study, we showed that traits of high cognitive ability and long educational duration, as well as traits of tall height and overweight or obesity in young adulthood, were associated with fewer cases of schizophrenia than the contrasting reference levels. These traits are shaped by early life environment, yet, it is important to notice that while cognitive abilities and body height are rather stable throughout most of adult life, BMI and educational duration may be subject to change. During adult lifespan, schizophrenia contributed around 1500 and 500 additional deaths of natural and unnatural causes per 100,000 py. Cognitive ability, educational duration, height, and BMI in young adulthood seemed to contribute equally to mortality from natural and unnatural causes in men with and without schizophrenia except for tall height, long educational duration, and obesity at conscription. Thus, long educational duration at conscription were associated with fewer deaths from natural causes and obesity was associated with fewer deaths from unnatural causes in men with schizophrenia than among men without.

Importantly, supplementary analyses of a sub-population of the most recent birth cohorts (the DCR) showed that the findings were comparable for the findings with and without adjustment for father’s education. Thus, we do not expect the main findings to be solely explained by social background during the men’s upbringing.

### Strengths and limitations

The study is based on a very large population followed from early adulthood until a maximum age of 75 years. The study includes objective measures of traits in young adulthood, and the outcomes schizophrenia and causes of death are obtained from register-based data. The traits were only measured once, and whereas cognitive ability, height and education are relative stable, weight might change considerably during the life course. Changes in these measures may contribute to mortality both in men with and without schizophrenia. However, it was not a part of the aim of the present study to examine the effect of changes in weight during adulthood for men with or without schizophrenia on mortality risk. The validity of the schizophrenia diagnosis in the Danish register has been examined in two case reviews, which both showed that around 97.5% of patients registered with a schizophrenia diagnosis fulfilled the ICD10 criteria for a schizophrenia diagnosis [22, 23] and around 70% obtained the same diagnosis at later psychiatric hospital admissions [24]. However, a small study of the schizophrenia ICD-10 diagnosis in the Danish Psychiatric Central Register reported 66% true positives and 40% sensitivity [25]. The majority of men (96%) in the present study diagnosed with schizophrenia in the National Patient Register also had a diagnosis in the Danish Psychiatric Central Register. However, we cannot preclude that some individuals were registered with different psychiatric diagnoses such as misuse or bipolar disorder later in life. Identification of natural and unnatural causes of death showed similar validity [26]. Finally, an additive hazard model was applied, which may be more relevant to clinical decision-making by identifying subgroups where the largest number of cases could potentially be prevented [21]. To identify such groups, measures of excess risk rather than relative risk are most relevant as high relative risk estimates may translate into small absolute numbers for rare outcomes. Furthermore, interactions based on the additive rather than the multiplicative scale showing deviations from the sum rather than the product of the risk estimates are similarly more useful when aiming at identifying the group where most cases can potentially be prevented. It is a limitation that the exclusion of individuals with schizophrenia at baseline and exclusion of those who died or emigrated before 1969 can cause potential selection bias for the oldest birth cohorts. Also, since information on body height, weight and cognitive ability was only available in the Danish Conscription Registers, women were not included in this study, as the women who attend conscription are a highly selected population. The generalizability of the findings to women may, thus, be very limited, as they have a later age of onset of schizophrenia and lower mortality [27], and the influence of the traits, e.g. BMI, on the risk of schizophrenia may differ between the genders [5]. Men with schizophrenia before conscription would most likely not have attended the conscription examination and men with diagnosed schizophrenia were excluded from the analyses of development of schizophrenia. Yet, some prevalent cases of schizophrenia may be included, especially for the oldest birth cohorts born 1939-49 who were age 20–30 years at baseline. For the younger birth cohorts in DCD born 1950-59 and DCR, schizophrenia was in approximately 10% of cases diagnosed before age 20 years and in approximately 30% of cases diagnosed before age 30 years. The incidence of early onset schizophrenia is comparable to previous research [28, 29]. Finally, our study does not allow us to draw causal conclusions.”

### Comparison with previous studies

Our findings of premorbid low cognitive ability, lower height, and underweight in young adulthood being associated with a higher risk of schizophrenia are in agreement with previous studies from Sweden [7, 8], Finland [30], and Denmark [5, 6]. These findings only partly correspond to the original observation of Kretschmer that most of his patients with schizophrenia were tall and thin [31].

In the previous studies, the relationship between traits and schizophrenia has been reported as relative risks, whereas we estimated excess risk expressed as absolute numbers. Our findings show that being underweight contributed most to the excess cases of schizophrenia even when adjusting for cognitive function, educational duration, and height. The impact of being underweight early in life has been explained in the thrifty psychiatric phenotype concept, implying that exposure to early life stressors, such as impaired growth, may have a higher risk due to disruption of normal brain development [32]. Tallness has been associated with optimal growth and higher socioeconomic position early in life [33], thus acting as a possible protective factor.

The excess mortality in persons with schizophrenia is well described and has traditionally been measured by mortality rate ratios or differences in life expectancy compared with the general population [9, 10]. Previous studies have shown a higher relative risk for death from unnatural causes rather than from natural causes [9–11]. This study provides estimates of the excess mortality risk and found that schizophrenia was associated with almost 1500 more deaths from natural causes and around 500 more deaths from unnatural causes per 100,000 py. The major difference between the absolute and relative risk estimates is due to deaths from natural causes being much more common than death from unnatural causes, which leads to greater relative risk estimates for unnatural causes than natural causes.

The factors that contribute to the excess risk of death in individuals with schizophrenia are less well established in the literature, but poor physical health, smoking and substance misuse, diabetes, and hypertension have been forwarded as the main risk factors of excess death in individuals with schizophrenia [2]. In our study, the four traits: cognitive ability, educational duration, height, and BMI seemed to contribute equally to death from natural and unnatural causes in men with and without schizophrenia, except for long educational duration and obesity, where the first was more protective of death from natural causes, and the latter was more protective of death from unnatural causes among men with schizophrenia. It is plausible that long educational duration is a marker of a high degree of resources and less severe illness, which make patients cope better with their disease and thereby prolong survival. Furthermore, death from unnatural causes is a marker of the most severe and treatment-resistant psychiatric illnesses [34, 35]. Our finding that especially premorbid obesity seems to be a trait associated with lower risk of death from unnatural causes in men with schizophrenia accords with previous findings. A study French study of 3470 patients meeting the ICD-10 criteria for schizophrenia showed that lower BMI was associated with higher risk of mortality by suicide [36]. Furthermore, three large studies of 630,807 to 1,133,019 Danish and Swedish men have shown inverse associations between BMI and risk of depression (stable from BMI > 25) and bipolar disorder as well as death by suicide in the general population of men [37–39]. The association may be reinforced in men with schizophrenia due to the larger proportion of death by unnatural causes compared to the background population. However, it is important to also notice that previous studies have shown associations between obesity and suicide behavior, mood disorder and anxiety [40–42], which contradicts our findings. Yet, this may be explained by differences in study design and outcome measures by death by unnatural causes versus suicide behavior, mood disorders and anxiety.

## Conclusion

Traits of high cognitive ability and long educational duration, tall height, and overweight or obesity are associated with a lower absolute number of incident cases of schizophrenia in men. Interactions were identified between some of these traits and schizophrenia by the trait of long educational duration being associated with fewer deaths from natural causes, and the trait of obesity being associated with fewer deaths from unnatural causes among men with schizophrenia. The finding of a lower risk of death from unnatural causes associated with obesity in men with schizophrenia is in accordance with the lower risk of suicide in individuals with overweight or obesity that may be reinforced in men with schizophrenia. Sub-analyses of the most recent cohorts showed that adjustment for father’s educational duration did not change the findings. The associations between schizophrenia, each of the four traits and subsequent mortality found in this study open up for a number of possible explanatory factors such as genetic factors, early environmental factors and early illness factors (prodromal symptoms), which could be interesting to further explore in future studies to elucidate the underlying causal mechanisms.

## Electronic supplementary material

Below is the link to the electronic supplementary material.

Supplementary Material 1
