# Supplementary material for: Cognitive ability, education, height and body mass index in relation to risk of schizophrenia and mortality following its diagnosis
Source: Eur J Epidemiol. 2024 Jul 27;39(8):893–904. doi: 10.1007/s10654-024-01140-6 (PMC11410868; doi:10.1007/s10654-024-01140-6)
Supplement: Supplementary file 1 — Supplementary Material 1 [file 10654_2024_1140_MOESM1_ESM.docx]

**Supplementary file**


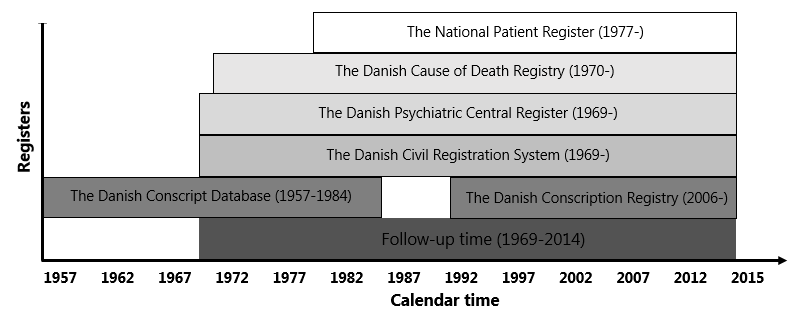


**Supplementary Fig. S1** Timeline of data collection from each of the registers and the total follow-up time


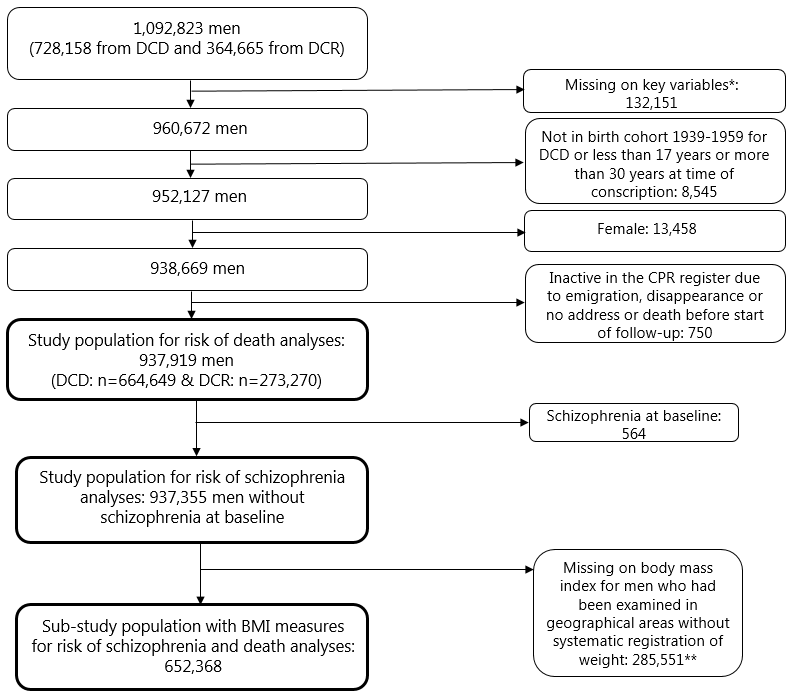


**Supplementary Fig. S2** Selection of the study populations in bold circles

* Missing due to lack of information for those exempted from the draft board examination due to medical conditions such as mental retardation, asthma, epilepsy, or type 1 diabetes

** In previous papers, we have shown that men with missing information on BMI do not represent at selective part of the study population (Jørgensen et al., 2016).

Abbreviations: DCD: Danish conscription database, DCR: Danish conscription Register, CPR: the Danish civil registration system


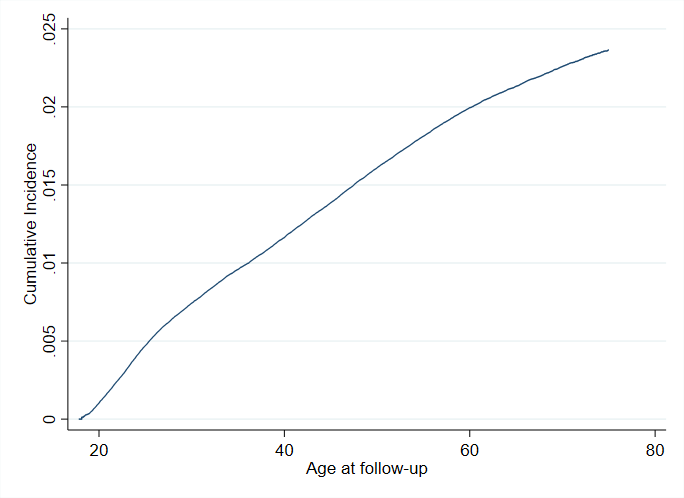


**Supplementary Fig. S3** Nelson-Aalen cumulative hazard curves of schizophrenia


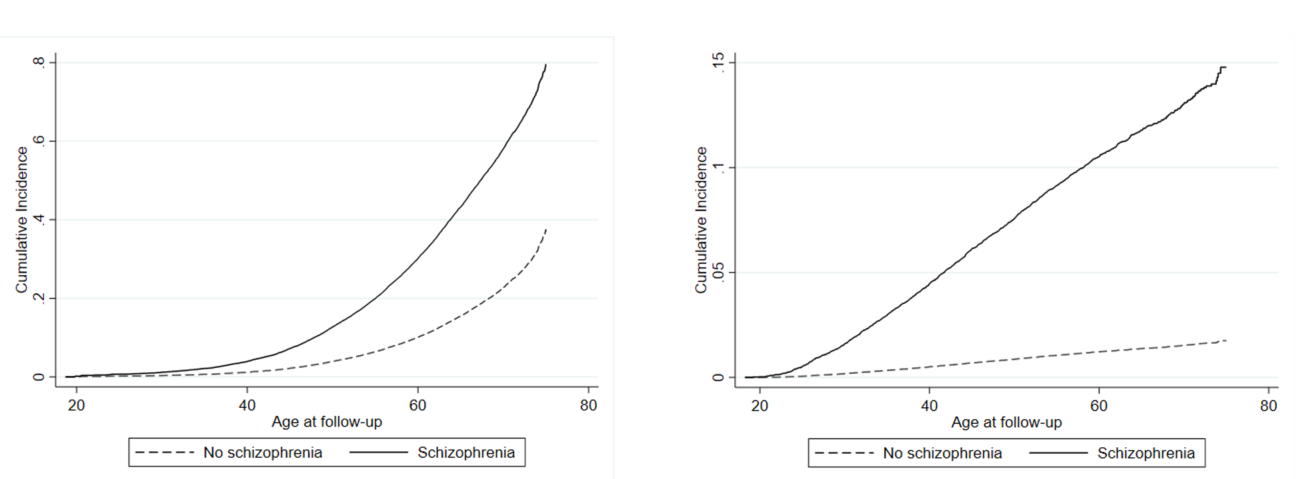


**Supplementary Fig. S4** Nelson-Aalen cumulative hazard curves of death from natural causes (left) and unnatural causes (right) for men with and without schizophrenia based on the match cohort of men with and without schizophrenia

| **Supplementary Table S1** Baseline characteristics in the full study population, the 1:5 matched sample used in the cumulative risk analysis and among men who had or develop schizophrenia | | | |
| --- | --- | --- | --- |
|  | Number of men  (%) | Number of men in the 1:5 matched sample  (%) | Number with schizophrenia (%) |
| **Total**  Schizophrenia at baseline  Schizophrenia during follow-up | 937,919 (100.0) | 61,300 (100.0) | 12,882 (100.0)  564 (4.4)  12,318 (95.6) |
| **Cognitive ability**  Lowest tertile  medium tertile  Highest tertile  Mean [SD] | 316,368 (33.7)  339,770 (36.2)  281,781 (30.0)  38.8 [11.4] | 21,389 (34.9)  21,463 (35.0)  18,448 (30.1)  38.7 [11.6] | 6,048 (47.2)  3,934 (30.7)  2,840 (22.2)  34.8 [12.7] |
| **Educational duration**  Short  medium  Long | 243,617(26.0)  462,014(50.0)  223,008(24.2) | 15,732 (25.7)  33,019 (53.9)  12,549 (20.5) | 4,858(37.9)  5,719 (44.7)  2,245(17.5) |
| **Height (cm)**  Lowest tertile  medium tertile  Highest tertile  Mean [SD] | 346,712 (37.0)  318,488 (34.0)  272,719 (29.1)  177.8 [6.8] | 22,349 (36.5)  19,71 (32.2)  19,230 (31.4)  177.5 [6.7] | 5,384 (42.0)  4,231 (33.0)  3,207 (25.0)  177.0 [6.8] |
| **Sample with BMI** | | | |
| **Total** | 652,368 | 39,235 | 9,534 |
| **Body mass index (kg/m2)**  Underweight  Normal  Overweight  Obese  Mean [SD] | 35,841 (5.5)  504,191 (77.3)  91,190 (13.8)  21,190 (3.2)  22.5 [3.2] | 2,300 (5.9)  504,191 (80.5)  91,190 (11.4)  21,190 (2.8)  21.5 [2.9] | 930 (9.8)  7,504 (79.4)  848 (8.9)  192 (2.0)  21.6 [3.0] |

| **Supplementary Table S2** Additional cases of schizophrenia per 100,000 person-years associated with the traits from mutually adjusted additive hazard models | | | |
| --- | --- | --- | --- |
|  | | Mutually adjusted additional cases of schizophrenia (95% CI) | |
|  | | DCR* | DCD |
| Full study population | | | |
| Cognitive ability | Low | Reference | Reference |
|  | Medium | **-35 (-49;-20)** | **-21 (-24; -17)** |
|  | High | **-32 (-46;-17)** | **-29 (-33;-25)** |
| Educational duration | Short | Reference | Reference |
|  | Medium | **-62 (-77;-48)** | **-23 (-27;-20)** |
|  | Long | **-114 (-127;-100** | **-6 (-11;-1)** |
| Height | Low | Reference | Reference |
|  | Medium | **-20 (-33;-8)** | **-4 (-7;-1)** |
|  | High | **-22 (-34;-11)** | **-6 (-9;-3)** |
| Study population including BMI measures | | | |
| Body mass index | Underweight | **76 (45;106)** | **38 (30;47)** |
|  | Normal | Reference | Reference |
|  | Overweight | **-22 (-34;-10)** | **-20 (-27;-17)** |
|  | Obese | -13 (-34;9) | **-40 (-50;-29)** |
| *The DCD cohort was restricted to the birth cohorts 1950-1959 and followed for their conscription examination and until 1978-80 to ensure comparable follow-up time in the two cohorts (N= 298,658) | | | |

| **Supplementary Table S3** Additional deaths per 100,000 person-years* associated with the exposure variables | | | | | |
| --- | --- | --- | --- | --- | --- |
|  | | Additional number deaths  from natural cause  (95% CI) | | Additional number  deaths  from unnatural causes  (95% CI) | |
|  | | DCR | DCD* | DCR | DCD* |
| Schizophrenia |  | **-8 (-11;-4)** | **1240 (1070;1400)** | 69 (-4;2) | **556 (448;664)** |
|  | | | | | |
| Cognitive ability | Low | Reference | Reference | Reference | Reference |
|  | Medium | -2 (-4;1) | **-64 (-71;-57)** | **-9 (-14;-3)** | **-12 (-15;-8)** |
|  | High | 1 (-2;5) | **-90 (-98;-83)** | **-9 (-14;-3)** | **-21 (-25;-16)** |
| Interaction between cognitive ability and schizophrenia | Low | Reference | Reference | Reference | reference |
|  | Medium | 2 (-1;4) | -46 (-240;148) | -5 (-147;138) | 89 (-47;224) |
|  | High | -2 (-5;2) | **-2 (-217;214)** | 4 (-2;2) | 101 (-57;258) |
|  | | | | | |
| Educational duration | Short | Reference | Reference | Reference | Reference |
|  | Medium | -2 (-5;1) | **-88 (-96;-81)** | **-16 (-22;-11)** | **-43 (-47;-38)** |
|  | Long | **-5 (-7;-2)** | **-130 (-140;-120)** | **-22 (-27;-17)** | **-55 (-61;-50)** |
| Interaction between education and schizophrenia | short | Reference | Reference | Reference | reference |
|  | Medium | 2 (-8;5) | -113 (-296;70) | 106 (-56;269) | 2 (-121;125) |
|  | Long | **5 (2;8)** | **-423 (-657;-190)** | 200 (-28;428) | -80 (-251;91) |
|  | | | | | |
| Height | Low | Reference | Reference | Reference | Reference |
|  | Medium | -0 (-3;2) | **-31 (-38;-24)** | -4 (-8;1) | **-11 (-15;-7)** |
|  | High | -4 (-3;2) | **-37 (-43;-30)** | -3 (-7;2) | **-16 (-20;-12)** |
| Interaction between height and schizophrenia | Low | Reference | Reference | Reference | Reference |
|  | Medium | 0.2 (-2;3) | 51 (-119;221) | -119 (-259;21) | -7 (-131;117) |
|  | High | 0.1 (-2;3) | -39 (-217;138) | -18 (-205;169) | **-130 (-257;-3)** |
| Study population including BMI measures | | | | | |
| Schizophrenia |  | **-7 (-10;-5)** | **1220 (1020;1420)** | 51 (-270;129) | **595 (458;732)** |
|  | | | | | |
| Body mass index | Underweight | -2 (-6;2) | **78 (64;93)** | 5 (-6;15) | **14.1 (6;2)** |
|  | Normal | Reference | Reference | Reference | Reference |
|  | Overweight | -2 (-4;1) | **31 (16;46)** | -2 (-7;2) | **-16 (-23;-10)** |
|  | Obese | 4 (-3;10) | **168 (122;214)** | -6 (-13;2) | **-18 (-35;-0)** |
| Interaction between body mass index and schizophrenia | Underweight | 2 (-2;6) | 147 (-146;441) | 0 (-276;276) | 43 (-147;233) |
|  | Normal | Reference | Reference | Reference | Reference |
|  | Overweight | 2 (-1;4) | 279 (-128;687) | -1 (-213;51) | -54 (-295;187) |
|  | Obese | -4 (-10;3) | 494 (-950;1940) | **-130 (-219;-42)** | -127 (-821;567) |
| *The DCD cohort was restricted to the birth cohorts 1950-1959 and followed for their conscription examination and until 1978-80 to ensure comparable follow-up time in the two cohorts (N= 298,658) | | | | | |
